# Insights to enhance the examination of tool marks in human cartilage

**DOI:** 10.1007/s00414-021-02609-9

**Published:** 2021-05-13

**Authors:** Matthias Weber, Anja Niehoff, Markus A. Rothschild

**Affiliations:** 1 Institute of Legal Medicine, Faculty of Medicine, University of Cologne, Melatengürtel 60-62, 50823 Cologne, Germany; 2 Institute for Forensic Sciences, Landeskriminalamt Nordrhein-Westfalen (LKA NRW), 40219 Düsseldorf, Germany; 3 Institute of Biomechanics and Orthopaedics, German Sport University Cologne, 50933 Cologne, Germany; 4 Cologne Center for Musculoskeletal Biomechanics (CCMB), Medical Faculty, University of Cologne, 50931 Cologne, Germany

**Keywords:** Tool marks, Cartilage, Cross-correlation, Casting, Striations, Sharp force trauma

## Abstract

This work deals with the examination of tool marks in human cartilage. We compared the effectiveness of several cleaning methods on cut marks in porcine cartilage. The method *cleaning by multiple casts* achieved the significantly highest scores (*P* = 0.02). Furthermore, we examined the grain-like elevations (dots) located on casts of cut cartilage. The results of this study suggest that the casting material forms these dots when penetrating cartilage cavities, which are areas where the strong collagen fibres leave space for the chondrocytes. We performed fixation experiments to avoid this, without success. In addition, 31 casting materials were compared regarding contrast under light-microscope and 3D tool marks scanner. Under the light-microscope, brown materials achieved significantly higher values than grey (*P* = 0.02) or black (*P* = 0.00) whereas under the 3D scanner, black materials reached higher contrast values than grey (*P* = 0.04) or brown (*P* = 0.047). To compare the accuracy and reproducibility of 6 test materials for cartilage, we used 10 knives to create cut marks that were subsequently scanned. During the alignment of the individual signals of each mark, the cross-correlation coefficients (X_max_) and lags (L_Xmax_) were calculated. The signals of the marks in agarose were aligned with significantly fewer lags and achieved significantly higher cross-correlation coefficients compared to all tested materials (both *P* = 0.00). Moreover, we determined the cross-correlation coefficients (X_C_) for known-matches (KM) per material. Agarose achieved significantly higher values than AccuTrans®, Clear Ballistics™, and gelatine (all *P* = 0.00). The results of this work provide valuable insights for the forensic investigation of marks in human costal cartilage.

## Introduction

The forensic examination and evaluation of tool marks was first mentioned in 1893 by the Austrian judge Hans Gross^1^ [1], who advised that marks at burglary scenes should always be closely examined, drawn or cast. In 1900, the forensic pathologist Kockel^2^ [2, 3] analyzed tool marks on cut trees in cases of vandalism “where young street trees have fallen victim to the exuberance of raw people”. Kockel compared the cut marks with test marks made with the knife of the suspect. He formulated the need for a homogeneous and opaque test material and used gypsum boards to generate test marks in it. To create even test marks, Kockel used a microtome slide, and by comparing the cut marks and test marks, he was able to identify the knife responsible for cutting the trees. Kockel documented his findings using photography. Only a short time later, Bischoff [4] conducted similar examinations and came to the same conclusions.

The comparative tool marks analysis was applied by Esser [5], Bosch [6] and Bonte [7–9] to marks resulting from sharp force trauma on human tissue, and is now performed on human bone [10–14] and cartilage [15–17] with the aim of identifying or excluding the suspect’s weapon or tool. Even tool marks in soft tissue are examined and at least class characteristics of the weapon or tool can be determined [18, 19].

**Fig. 1:**
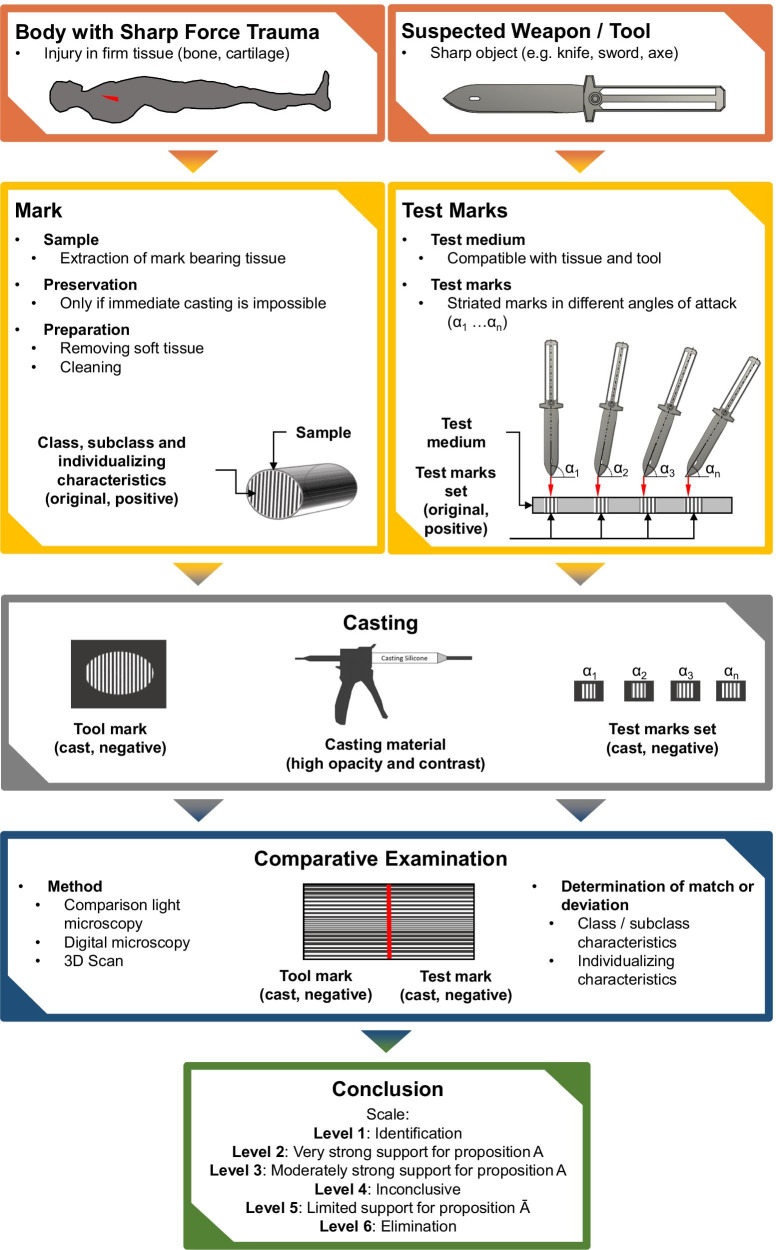
Schematic representation of the examination process of tool marks in human tissue. The top level (red) shows the securing of marks and murder weapon/tool. The level below (yellow) shows the preparation of marks and test marks. The following levels show the production of casts (grey) and the comparative examination (blue). The last level represents the finding of the conclusion (green)

The examination of tool marks on human tissue is regularly performed in the following steps (Fig. 1): Collecting mark-bearing samples, preservation and preparation of the tissue, production of test marks with the tools or weapons in question, casting of marks and test marks, comparative examination and conclusion [20]. No or very little research has been done on several of these steps. After collecting the mark-bearing specimens, it may be necessary to store them if timely casting is not possible. Stanley et al. [21] demonstrated the effects of decay on striated stab wounds under various conditions. Wong [22] researched various methods for the preservation of tool marked cartilage and bone tissue and suggested to submerge the specimens in 0.9% NaCl saline and immediately freeze them. King et al. [23] describe microwave heating as a fast, effective, and comparatively clean method for macerating bones. Weber et al. [14] macerated striated marks bearing bone by simmering in a solution of water and washing powder at 75 °C and found no differences on the topography of the marks before and after maceration. As an alternative for dry storage of bone Bailey et al. [24] suggest to store the specimens in antimicrobial solutions.

Compared to technical materials, but also compared to bones, cartilage places higher demands on tool marks analysis. While the casting of macerated bone is analogous to the casting of marks on technical materials, tissue residues and contamination can significantly complicate the securing of marks on cartilage samples.

To our knowledge, no research has been done concerning the preparation (cleaning, removing of soft tissue) of mark bearing cartilage tissue before casting. A recommendation in the literature is to degrease the marks with alcohol [25].

Although the use of silicone-based casting materials has been described in numerous studies and case reports [15, 17, 26–28], no work exists in which such materials are comparatively examined for their applicability in toolmark examination and, in particular, with regard to their use under the light microscope and the tool mark scanner.

Furthermore, only little work was done on the selection of a cartilage analogous test material, which is critical for a comparative tool mark examination. Generally speaking, a test material must meet some essential requirements: It needs to be soft enough not to alter the tool surface [29]. It must be able to show very fine details; in the case of stab and cut marks, this would be very fine striations. It must be possible to reproduce the same mark under the same conditions. Ideally, the test material should also be non-toxic and safe to handle and, in the best case, inexpensive. The use of animal cartilage as test material is not possible because the material is too inhomogeneous and the dimensions are not sufficient to produce test marks at all the necessary angles. Dip-Pak®,^3^ a cellulose-based coating material, and ballistic gelatine are used as standard test materials for cut marks in human costal cartilage [15–17, 30, 31]. In a survey, the authors of this study asked 12 institutes that carry out tool marks analysis on cartilage in which test materials are in use. The answers given included casting material, Transresin^4^ bone cutting plates, Clear Ballistics™^5^ synthetic ballistics gelatine, vinyl, rubber and soft plastic. However, no comparative study of test materials for marks in cartilage has been presented so far.

The purpose of this work is to address some of these unsolved questions. At first, we aimed to improve the quality of the casts of tool mark bearing cartilage by identifying an effective way for cleaning the tissue, which is, in most cases, contaminated with greasy and bloody residues.

Casts of cut marks in rib cartilage may show sand grain-like elevations, referred to as “dots” in the following. Even though these dots have not been mentioned in any study to date, they are nevertheless recognizable in the figures of numerous studies [16, 22, 26, 32–34]. We hypothesize that the dots occur when casting material enters and fills the lacunae of the chondrocytes. To investigate this further, we compared the size and distribution of dots and chondrocytes. In addition, we investigated if these disturbances can be avoided by fixation of the cartilage tissue. Porcine rib cartilage specimens were submerged in different solutions, and cut marks in the tissue were cast both before the fixing procedure and afterwards. We also tested if fixing the cartilage before producing the cut marks affects the occurrence of dots.

Moreover, we compared 31 casting materials by analyzing the contrast of light microscopic images and scan images. The aim was to identify materials that show the highest contrast and are, therefore, best suited for the tool marks analysis.

With the goal of finding a more suitable test material for the comparative examination of tool marks in cartilage, we examined 6 potential materials with regard to the quality and reproducibility of cut marks. To this end, we compared the signatures of three-dimensionally scanned cut marks by comparing their cross-correlation [35–37] as well as the displacement (lag) required in preprocessing. In addition, we determined the Young’s Modulus of the materials by indentation testing and compared the results with human rib cartilage.

## Method

### Cleaning of costal cartilage

**Fig. 2:**
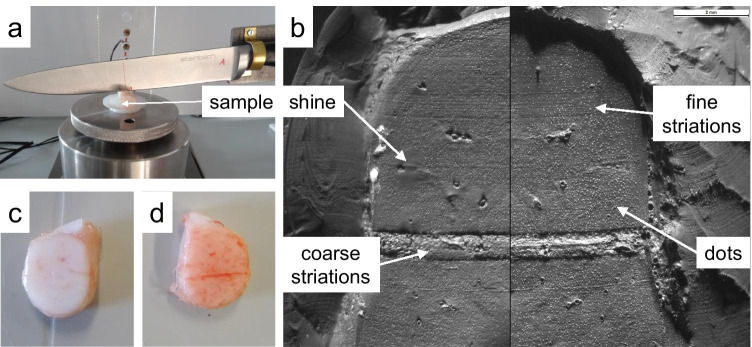
(**a**) Cutting of a cartilage sample for the cleaning experiments. (**b**) Microscopic comparison of casts of a mark (right) and the reference mark (left). (**c**) Reference sample (not contaminated). (**d**) Contaminated and cleaned sample

Due to the limited amount of body donors, porcine rib cartilage (*N* = 75 samples) was used for this series of tests instead of human cartilage samples. The frozen cartilage samples were thawed at room temperature approx. 1 h prior to the experiments. Using a custom-made cutting device consisting of a single non-serrated knife (Steinbach kitchen knife, blade length 205 mm) fixed on a manually moveable lever, reproducible cuts were produced in the samples (Fig. 2).

**Fig. 3:**
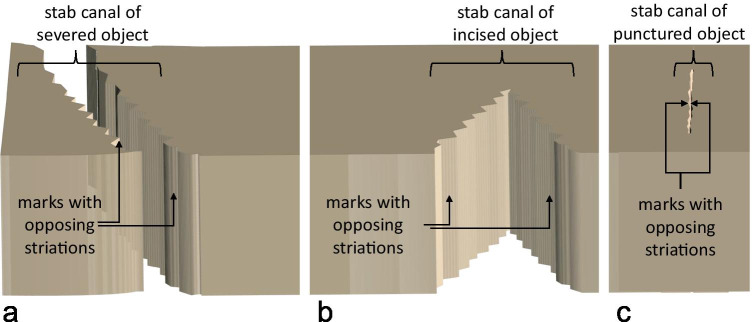
Schematic of stab/cut canals resulting from severance (**a**), incision (**b**) and puncturing (**c**) of an object with a blade. All canals consist of marks with opposing striations

A cut or stab with a knife in rib cartilage can either sever it completely, incise or puncture it. Each case creates a canal in the cartilage whose sidewalls consist of striated marks invers to each other (Fig. 3). A cut in this context describes the separation of material with the cutting edge facing forward, while a stab describes a tip-first motion. Since cut and stab canals in cartilage consist of two marks with opposing striations [6, 7, 38], one mark per canal was used for the cleaning experiment and the matching mark was used as reference (75 reference samples). The marks were contaminated by brushing up either porcine blood, porcine fat or an emulsion of both. Afterwards, the contaminated marks were cleaned with one of the following methods: (Cool) Water, ethanol, tri buffered saline (TBS), TBS mixed with 1% detergent (tween® 20^6^) or triple casting with AccuTrans®^7^ AB brown. For this purpose, the fluids were carefully rubbed over the mark surface using a sponge. Five marks and 5 reference marks were produced for every combination of contamination and cleaning method. Afterwards all cleaned marks and untreated reference marks were cast using AccuTrans® AB brown and comparatively examined using a Leica FS-C Light microscope. Scores were assigned according to the criteria coarse striations detectable, fine striations detectable, complete mark detectable, no shine, no dots. One score per combination was awarded for fulfilling the category and 0 score for not fulfilling the category. For the category no dots also -1 scores were assigned if more dots occurred compared with the reference mark. For every cleaning method the total scores (TS) for the samples and the reference samples were calculated. In order to determine the effects of the cleaning methods, the difference between the two values was calculated as delta score (DS).

### Cartilage dots

The dots on castings of cut human costal cartilage of 3 bodies (all female, mean age 32.7 + / − 6.5 years) were microscopically (digital microscope: Keyence VHX-2000; Osaka, Japan) examined and the diameters D_C_ of ten dots per cast (total of 30) were measured. In addition, hematoxylin–eosin-stained sections (automated stainer: Sakura DRS 2000 automated slide stainer) of 4 bodies (all male, mean age 54.5 + / − 23.4 years) were microscopically examined and the diameters D_H_ of ten chondrocytes per section (total of 40) were measured.

At − 20 °C 36 frozen samples of porcine costal cartilage were thawed in NaCl 0.9% solution at room temperature for approx. 1 h and cut marks were produced using a kitchen knife (Steinbach kitchen knife, blade length 205 mm). Preliminary tests to determine the suitability of the potentially applicable fixatives formaldehyde, paraformaldehyde and glutaraldehyde have shown that glutaraldehyde leads to significant tissue changes or crystallization of tissue components after a short exposure time of < 30 min and is therefore unsuitable. The experiments were carried out with 10% formaldehyde and 4% paraformaldehyde. Three samples were submerged in the fixatives for T = 30 min, 1 h, 2 h, 4 h or 24 h and afterwards casts of the cut marks on all samples were made and microscopically analyzed.

To assess whether fixing before the cutting process would prevent the appearing of dots, samples were at first submerged in 10% formaldehyde or 4% paraformaldehyde for T = 90 h. The high exposure time compared to the previous experiments was chosen to ensure complete fixation of the tissue prior to cutting. Subsequently, all samples were cut with the previously used kitchen knife and the marks were cast and microscopically examined.

### Contrast of casting materials

Aiming to determine their suitability for tool marks analysis, the contrast values of light microscopic images and 3D tool marks scanner images of 31 casting materials (Table 1) were determined. Samples were taken by casting the striation pattern of three roughness standards (Halle Feinwerktechnik, Germany — roughness standards 1 fine, 2 middle, 3 rough). The colours of the materials were determined visually. In addition, the colour and light reflectance values LRV of the samples were determined by optical comparison with a RAL K7 colour fan deck. The LRV describes the proportion of light reflected by a surface regardless of how much or how little the surface is illuminated. A value of 100 stands for an ideally white surface, 0 for an ideally black one.

**Table 1:**
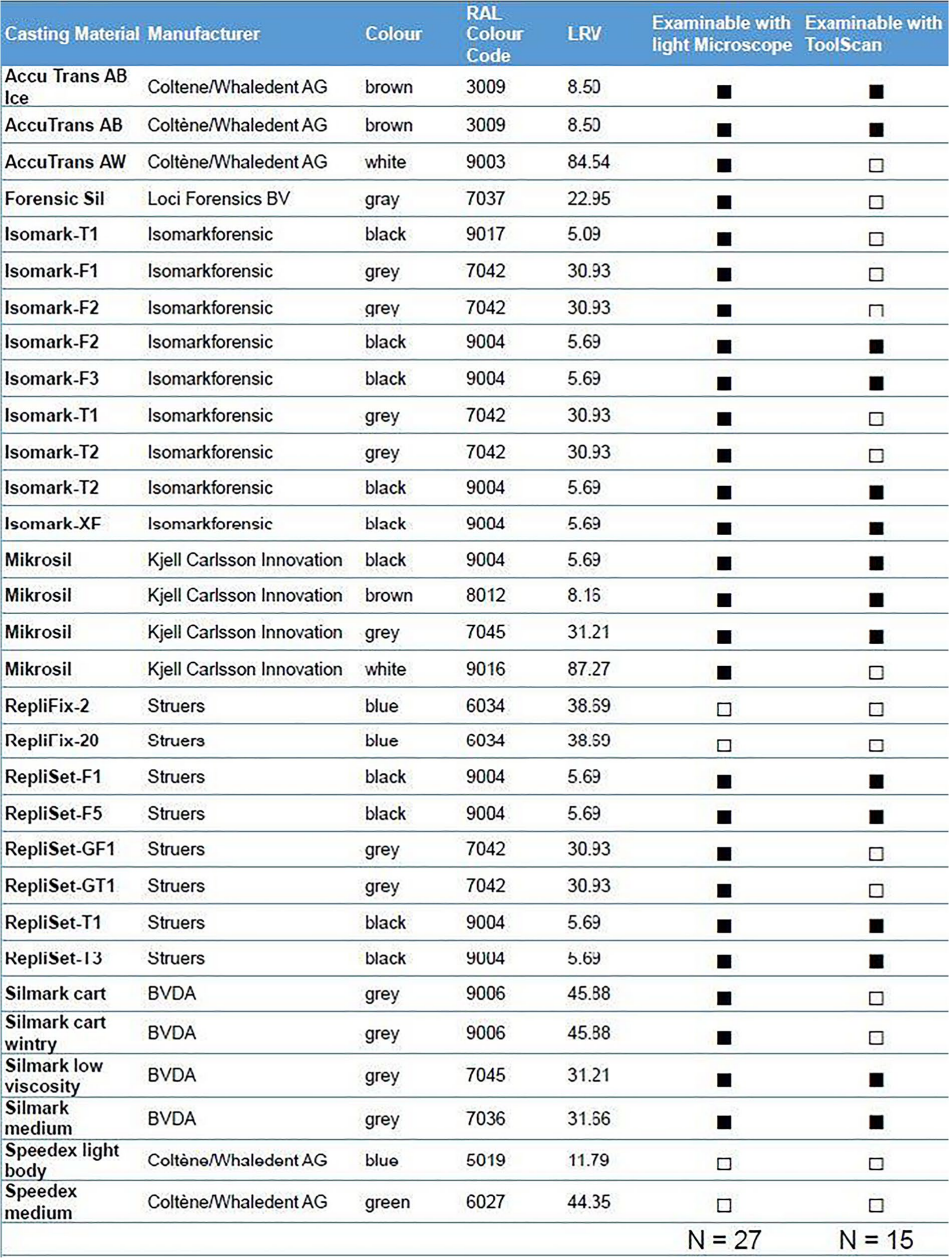
Listing of the 31 impression materials tested, colors, RAL colors and LVR (https://www.ral-farben.de, 26.12.2020) and examinability under light microscope and ToolScan (□ = no, ■ = yes)

Subsequently, light microscopic images of the samples were generated using a FS-C forensic comparison macroscope (Leica Microsystems) under oblique illumination (azimuth 0° perpendicular to the grooves, elevation angle 5°) and saved in JPG format with the following settings: Amplification = 1, saturation = 1.5, gamma = 0.6 (software: Leica LAS—version 4.12.0). The exposure time was manually adjusted to the brightness of the material.

Additionally, 3D scans were taken with a ToolScan 3D tool mark scanner (LIM Laboratory Imaging) that combines coarse surface features, which are determined with a laser scan of the surface, with detailed features determined from a series of circumferentially illuminated images in a resolution of 3 μm/px. Using the ToolScan software application (Version: 8.00) 3D representations of the scans were virtually illuminated (azimuth 0° perpendicular to the grooves, elevation angle 21°) and 2D images with and without texture^8^ were saved as JPG files. The optimal illumination intensity was determined automatically by the software.

In the next step, the image data were imported into MATLAB (MathWorks, version: R2018a). Since a high number of different brightness values is equivalent with a high-contrast and detailed mark representation, we have defined the dispersion of the brightness values as image contrast IC.

Thus, for each image IC was determined as the standard deviation of the brightness values from 0 (black/dark) to 255 (white/light) of the individual pixels. With the number of pixels n and the brightness value b, IC is therefore calculated according to Eq. (1).

1 $$IC=\sqrt{\frac{\sum_{i=1}^{n}({b}_{i}-\stackrel{-}{b}{)}^{2}}{n-1}}$$

Equation (1): Calculation of the image contrast IC. $\stackrel{-}{\mathrm{b}}$ represents the mean brightness b of all pixels.

### Quality and consistency of cut marks in the test materials

**Fig. 4:**
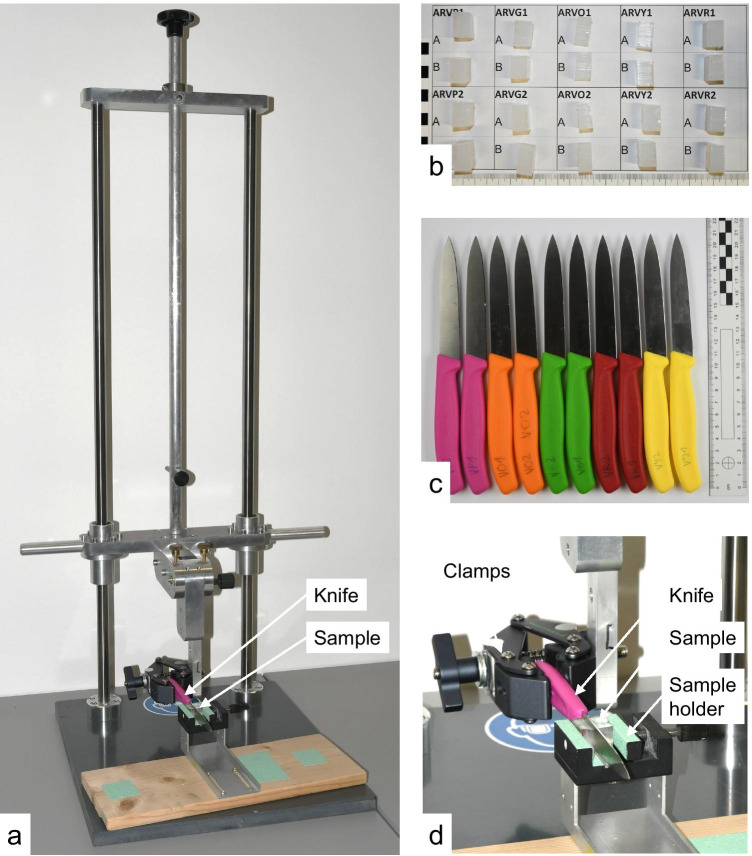
(**a**) Custom-made cutting device with recirculating ball bushings on two columns for exact vertical cuts. (**b**) Set of 20 agarose samples. The samples marked with A and B are known matches. (**c**) Ten Victorinox kitchen knives. (**d**) Detailed view of the cutting device showing the clamping of the knives and the sample holders

**Fig. 5:**
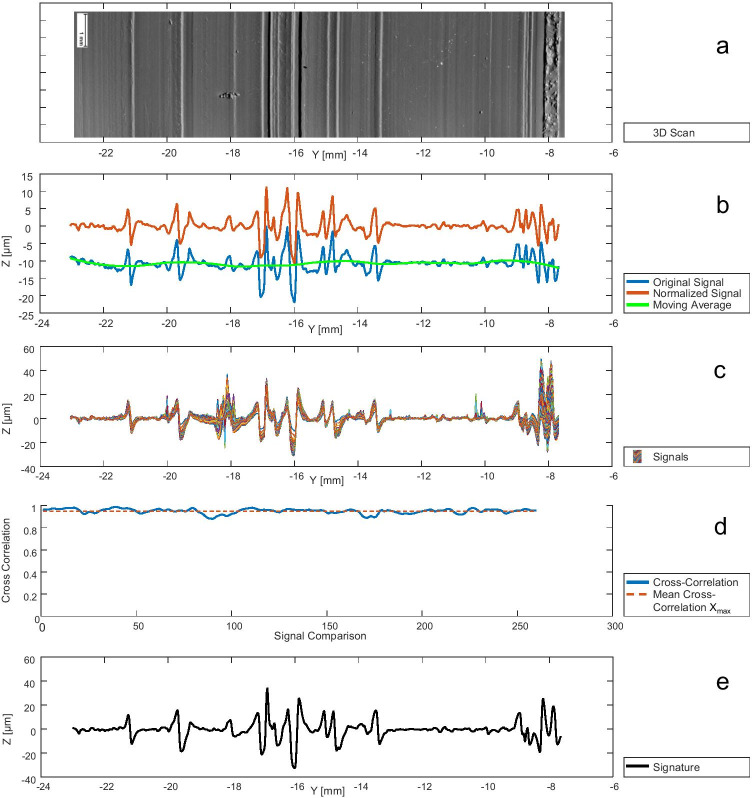
(**a**) Two-dimensional image of the 3D scan of the cast of a striated cut mark. (**b**) Exemplary representation of one (blue) of 261 signals to the mark shown above (a). The moving average (green) was subtracted from the blue signal to obtain the normalized signal (orange). (**c**) 261 signals associated to the mark shown above (a) after alignment. (**d**) Cross-correlation coefficients calculated in the preprocessing (alignment of the 261 signals). For every 260 comparisons the coefficient was calculated. The mean coefficient X_max_ is illustrated as orange dashed line. (**e**) The signature representing the mark shown above (a) calculated as the average of all 261 signals after alignment

In this study, the materials agarose^9^ 4%, Dip-Pak® (green), Clear Ballistics™, gelatine^10^ 20%, Trans Resin, and AccuTrans® AB brown were tested for suitability as test material for cut marks in human rib cartilage. For this purpose, 20 samples of each material of approx. 20 mm × 10 mm × 10 mm were prepared (Fig. 4). Ten identical kitchen knives (Victorinox Swiss Classic Paring Knife, item number 6.7703) were used to create cut marks in the samples. Two marks were generated per knife, so that 10 known matches (KM) and known non-matches (KNM) per material were available for the analysis. To ensure reproducible cutting, a custom-made device consisting of a holder in which the knives are clamped and vertical cuts can be made under manual force, was used. The surfaces of the cut marks were cast using AccuTrans® AB brown casting material with the exception of the cut mark created in this very material. The cut marks were then scanned using the previously mentioned ToolScan (Fig. 5a) and the 3D data was converted to STL files consisting of 262,044 points and imported into MATLAB. The marks in AccuTrans® AB brown casting material were not cast but scanned directly and the scan data were subsequently inverted.

To compare the test materials, we used the normalized cross-correlation method already successfully applied in other works for striation marks [36, 37, 39] where a correlation coefficient of 1 equals autocorrelation and 0 equals no correlation.

In the preprocessing, the first step was to divide the 262,044 data points per mark orthogonally to the cutting direction into S = 261 individual signals, each consisting of *P* = 1004 data points. The next step was the normalization, that is, to align each signal to the x-axis. This compensates for variations in topography that occur when the surface of the specimen is not aligned exactly parallel to the reference plane of the scanning device, or when the surface is uneven. For this purpose, the moving average Z-values were subtracted from each of the signals, discarding the low-frequency components (Fig. 5b). The high-frequency components caused by the fine cutting-edge structures of the knife blades were retained.

In the next step, the 261 signals per mark were aligned using normalized cross-correlation according to Eq. (2). The first two signals per mark were shifted and aligned to each other at the maximum cross correlation coefficient X_max_[1] with the lag L_Xmax_[1]. Subsequently, the third signal was shifted and aligned to the average of the first two already aligned signals at the maximum correlation coefficient X_max_[2] with the lag L_Xmax_[2], and so on. The signature representing the mark was then formed by averaging all 261 aligned individual signals (Fig. 5c). The mean of all maximum correlation coefficients X_max_ per mark was calculated to be a measure of the quality of the mark, where a high X_max_ close to 1.0 represents a high quality (Fig. 5d). The mean of all respecting lags L_Xmax_ was calculated as a measure of the straightness of the mark, where a higher value of L_Xmax_ represents a lower straightness since more shifting was necessary during the alignment of the 261 signals.

2 $$\mathrm{X}\left\{\mathrm{L}\right\}=\frac{\sum_{\mathrm{i}=1}^{\mathrm{p}}{\mathrm{S}}_{1}[\mathrm{i}]\bullet {\mathrm{S}}_{2}[\mathrm{i}+\mathrm{L}]}{\sqrt{\sum_{\mathrm{i}=1}^{\mathrm{p}}{({\mathrm{S}}_{1}\left[\mathrm{i}\right])}^{2}\bullet \sum_{\mathrm{i}=1}^{\mathrm{P}}{({\mathrm{S}}_{2}\left[\mathrm{i}+\mathrm{L}\right])}^{2}}}$$

Equation (2): Calculation of the normalized cross-correlation of the discrete signals S_1_ and S_2_ with a lag L; S_1_[i] and S_2_[i] are the vectors of the signals S_1_ and S_2_, p represents the number of discrete data points.

**Fig. 6:**
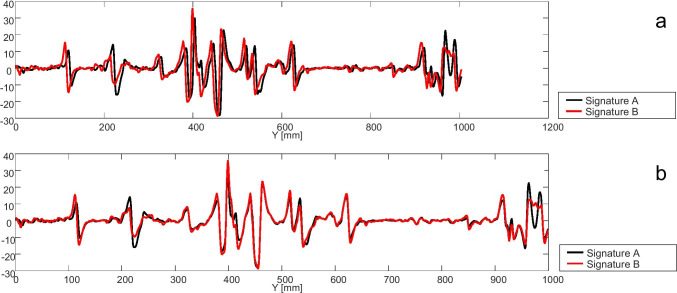
The signatures of two striated cut marks of a known match before (**a**) and after alignment (**b**). For this example, a cross-correlation coefficient of X_C_ = 0.93 was calculated

After the previously calculated alignment and averaging of the 261 signals per mark to one signature per mark (Fig. 5e) the comparison of the signatures (Fig. 6) of 10 known matches (KM) and 10 known non-matches (KNM) was performed for each test material using normalized cross-correlation (Eq. (2)). The cross-correlation coefficients during the comparison were named X_C_.

### Elastic properties of the test materials

**Fig. 7:**
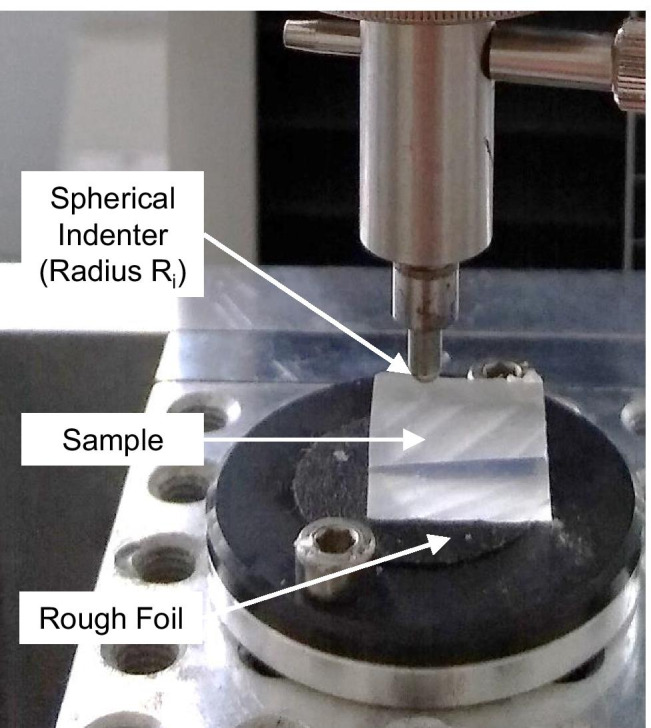
Experimental set-up of the indentation test. A custom made rigid spherical-tip (steel, radius R_i_ = 1.5 mm) is used as indenter. The samples are placed on a rigid plate below the indenter. To prevent the samples from slipping the surface of the plate is covered with a coarse foil

In order to determine the elastic material properties of the test materials agarose 4%, Dip-Pak® (green), Clear Ballistics™, gelatine 20%, Trans Resin, and AccuTrans® AB brown, indentations were carried out analogously to tests on human rib cartilage in a previous work [40]. The test setup was a desktop-type, single-column universal materials testing machine (Zwick BZ2.5/TN1S) with a 100 N force sensor and a spherical indenter (Fig. 7). A preload of 0.1 N was applied with a velocity of 0.05 mm/s. Afterwards, the displacement and force were set to 0 and the indenter was lowered with a velocity of 0.5 mm/s until the maximum indentation depth h_max_ of 0.9 mm was reached. Force, time, and displacement were measured at a sampling rate of 50 Hz. All data were imported into MATLAB (R2018a). The Young’s Modulus of indentation E_i_ was calculated according to Eq. (3) with the force F, the Poisson’s ratio $\upnu$ ($\upnu$= 0.5 for an incompressible solid [41–43]), the radius of the indenter *R* = 1.5 mm and the depth of indentation h_i_.

3 $${E}_{i}=\frac{3}{4}F\frac{{1-\nu }^{2}}{\sqrt{R}\bullet {h}_{i}^\frac{3}{2}}$$

Equation (3): Calculation of the Young’s Modulus E_i_ of indentation for the isotropic elastic Hertzian contact of a rigid spherical indenter and an incompressible material [44–46]*.*

### Statistics

All statistical analyses were performed using IBM ® SPSS ® Statistics Version 27 (IBM Corp. ®). A *P*-value below 0.05 was considered as statistically significant.

All test data of the cleaning experiments were checked with the Kolmogorov–Smirnov and Shapiro–Wilk tests and are not normally distributed (*P* < 0.05). The Wilcoxon-Test for dependent samples was used to determine for which cleaning method the total score TS of the cleaned samples differs significantly from the reference group. Afterwards the Mann–Whitney *U* test for independent samples was used to determine if the delta score DS of the methods “triple casting” and “TBS + Detergent” differ significantly.

The diameters of dots D_D_ and chondrocytes D_C_ were checked with the Kolmogorov–Smirnov and Shapiro–Wilk tests and are both distributed normally (*P* > 0.05). Both values were compared with the *t*-test for independent samples.

For the results of the light microscope, the data of the image contrast IC and the light reflectance value LRV were checked with the Kolmogorov–Smirnov and Shapiro–Wilk tests and are both not normally distributed (both *P* < 0.05). The values of IC and LRV were tested for linear correlation using the Pearson test. A possible effect of the colour on the IC value was checked using the Mann–Whitney *U* test for independent samples.

For the ToolScan, the data of the IC and the LRV were checked with the Kolmogorov–Smirnov and Shapiro–Wilk tests. While the IC is distributed normally (*P* = 0.20), this could not be detected for the LRV (*P* > 0.05). The values of IC and LRV were tested for linear correlation using the Pearson test. For the ToolScan, the influence of the LRV on the occurrence of faulty scans was investigated using the Mann–Whitney *U* test for independent samples.

The results of the mean cross-correlation coefficients X_max_ and the respective mean lag L_Xmax_ were checked with the Kolmogorov–Smirnov and Shapiro–Wilk tests and are both not distributed normally (both *P* < 0.05). For all test materials X_max_ and L_Xmax_ were compared using the Mann–Whitney *U* test for independent samples. The results of the cross-correlation coefficients X_C_ calculated in the comparison of the signatures of known-matches KM and known non-matches KNM were also tested with the Mann–Whitney *U* test for independent samples.

All results are presented as mean values ± standard deviation.

## Results

### Cleaning of costal cartilage

**Fig. 8:**
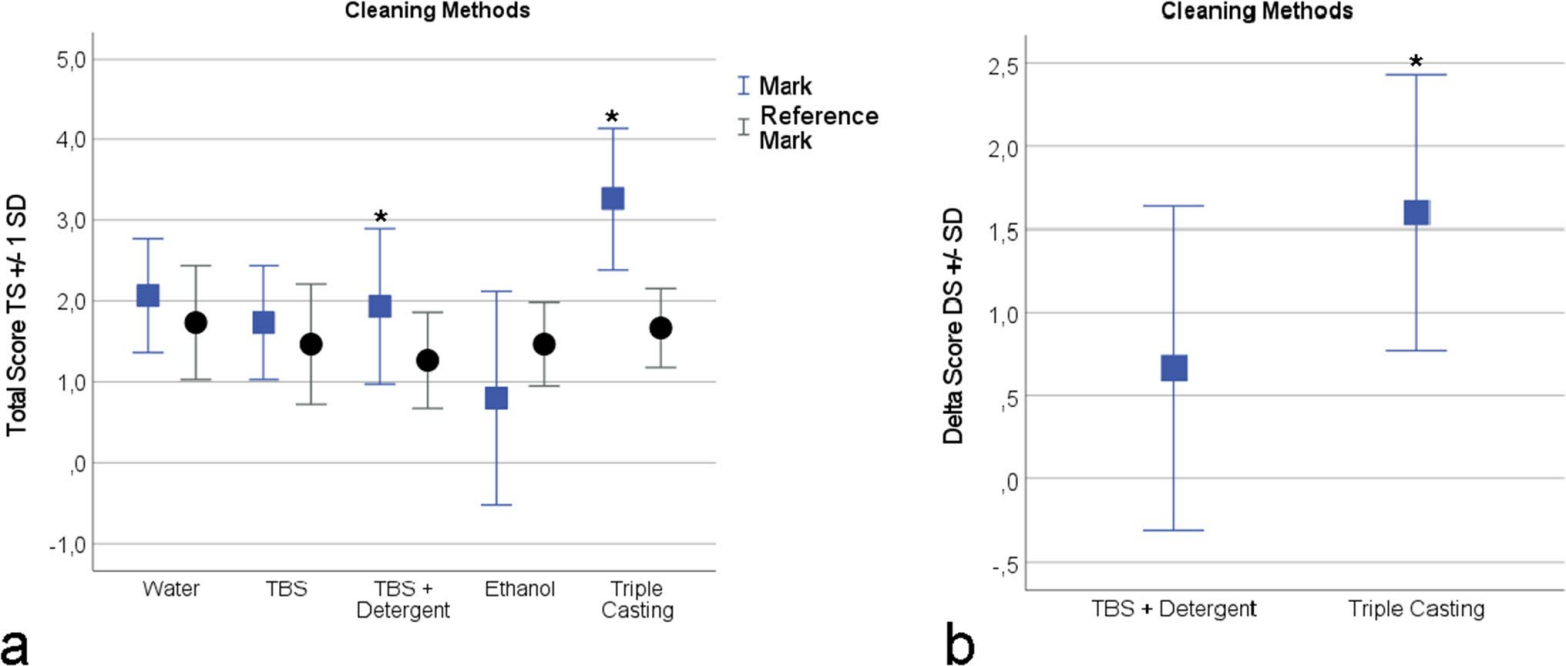
Results of the cleaning experiments. (**a**) The total score TS and TS_Reference_ per cleaning method. For “TBS + Detergent” and for “Triple Casting” the values of TS_Sample_ are significantly* higher than the values of TS_Reference_. (**b**) The delta score DS of the cleaning method “Triple Casting” yields significantly* higher scores then the DS of “TBS + Detergent”

The total scores TS of the cleaning methods *TBS* + *Detergent* and *Triple Casting* for the marks were both significantly higher (Fig. 8a) than the results for the respective reference group (for *TBS* + *Detergent*: TS = 1.93 + / − 0.96, TS_Reference_ = 1.27 + / − 0.59, *P* = 0.03; for *Triple Casting*: TS = 3.27 + / − 0.88, TS_Reference_ = 1.67 + / − 0.49, *P* = 0.00). By comparing the delta score DS of those two cleaning methods (Fig. 8b), it was found that the method *Triple Casting* yielded significantly higher results (*P* = 0.02).

### Cartilage dots

**Fig. 9:**
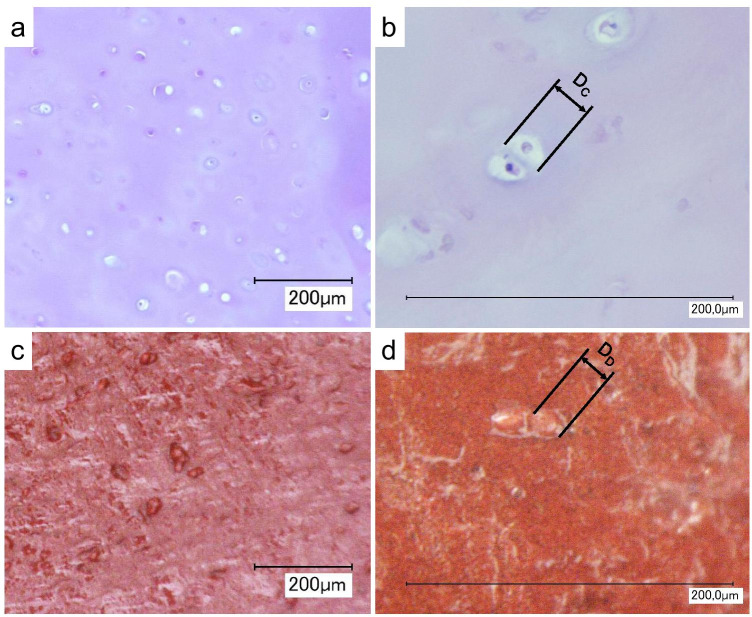
Microscopy images of HE stained sections of human costal cartilage (**a**, **b**) and casts of the cut surface of human costal cartilage (**c**, **d**)

By comparing the diameters (Fig. 9) of the dots (D_D_ = 26.3 + / − 5.1) and of the chondrocytes found on the HE stained costal cartilage sections (D_C_ = 29.2 + / − 7.6) no significant difference could be detected (*P* > 0.05). Furthermore, it was found that dots can be found individually and in groups of two and more. The usual grain like shape of the dots appears to be flattened for dots close to the perichondrium.

**Fig. 10:**
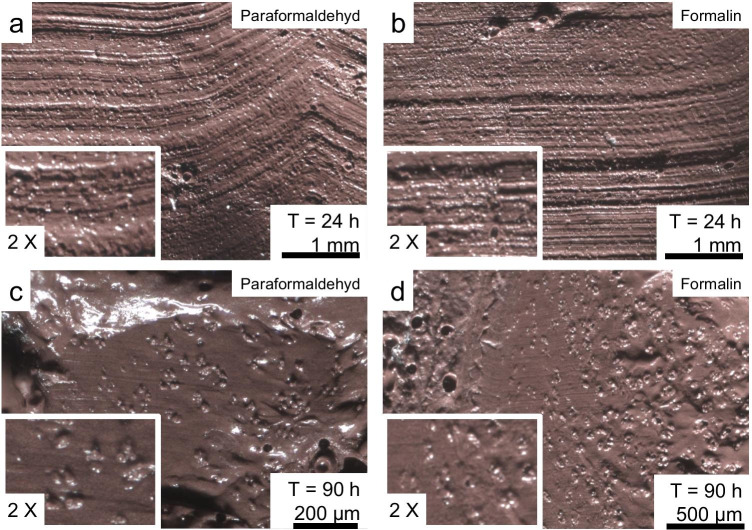
Dots on the casts of the fixation experiments. (**a**) and (**b**) show the casts of the samples first cut and afterwards submerged for 24 h into paraformaldehyde (**a**) or formalin fixation (**b**). Also, on the samples first submerged for 90 h into paraformaldehyde (**c**) or formalin (**d**) dots can be found

The fixation experiments on porcine cartilage specimens aiming to avoid the occurrence of dots were unsuccessful. Dots were detected on the casts of the samples that were first cut and then fixed, and on the samples that were cut and then cast after fixation (Fig. 10).

### Contrast of casting materials

When evaluating the images, it became apparent that some of the tested casting materials were not suitable for use on either the ToolScan or the light microscope or on both devices (Table 1). On the light microscope, one green and three blue samples were excluded, as they did not show any striation pattern, leaving 27 for further examination. On the ToolScan, samples that had obvious and pronounced mismeasurements, such as peaks and spikes, were classified as unusable including all blue, green, white, eight of the grey and one black material, leaving 15 usable materials.

**Fig. 11:**
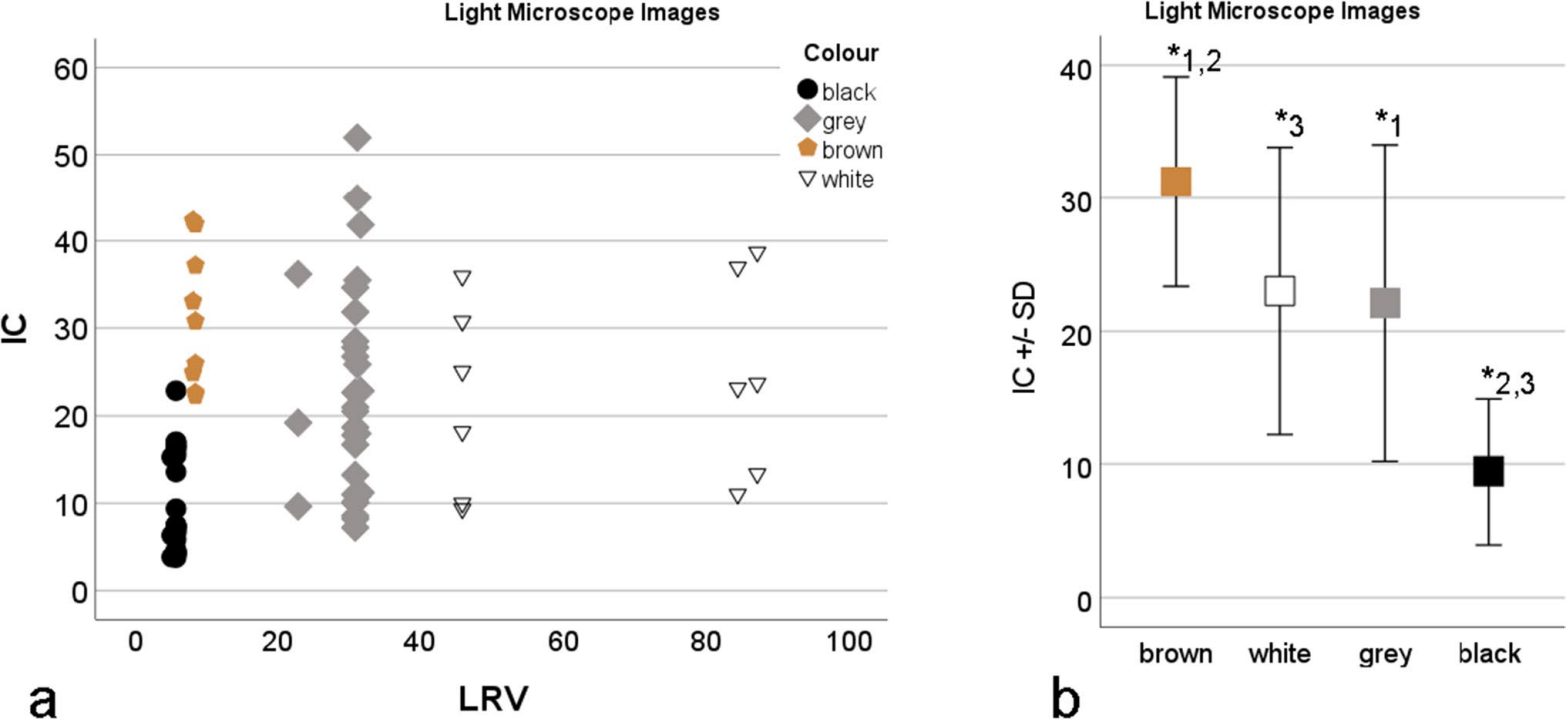
Results of the image contrast (IC) calculation for the light microscope. (**a**) Scatter plot of the IC over the light reflectance value (LRV) grouped by colour. (**b**) The effect of colour on the IC. Brown has significantly*^1,2^ higher values than grey and black. White shows significantly*^3^ higher results than black

The analysis of the results for the images of the light microscope revealed that the image contrast IC correlates significantly positive with the LRV (*R* = 0.31, *P* = 0.01, Fig. 11a). With regard to the colours of the samples, the IC values of the brown materials were found to be significantly higher than the values of the grey (*P* = 0.02) or black (*P* = 0.00) materials (Fig. 11b). The IC value of the white materials was significantly higher than the value of the black (*P* = 0.00). No other effects could be detected for the light microscope.

**Fig. 12:**
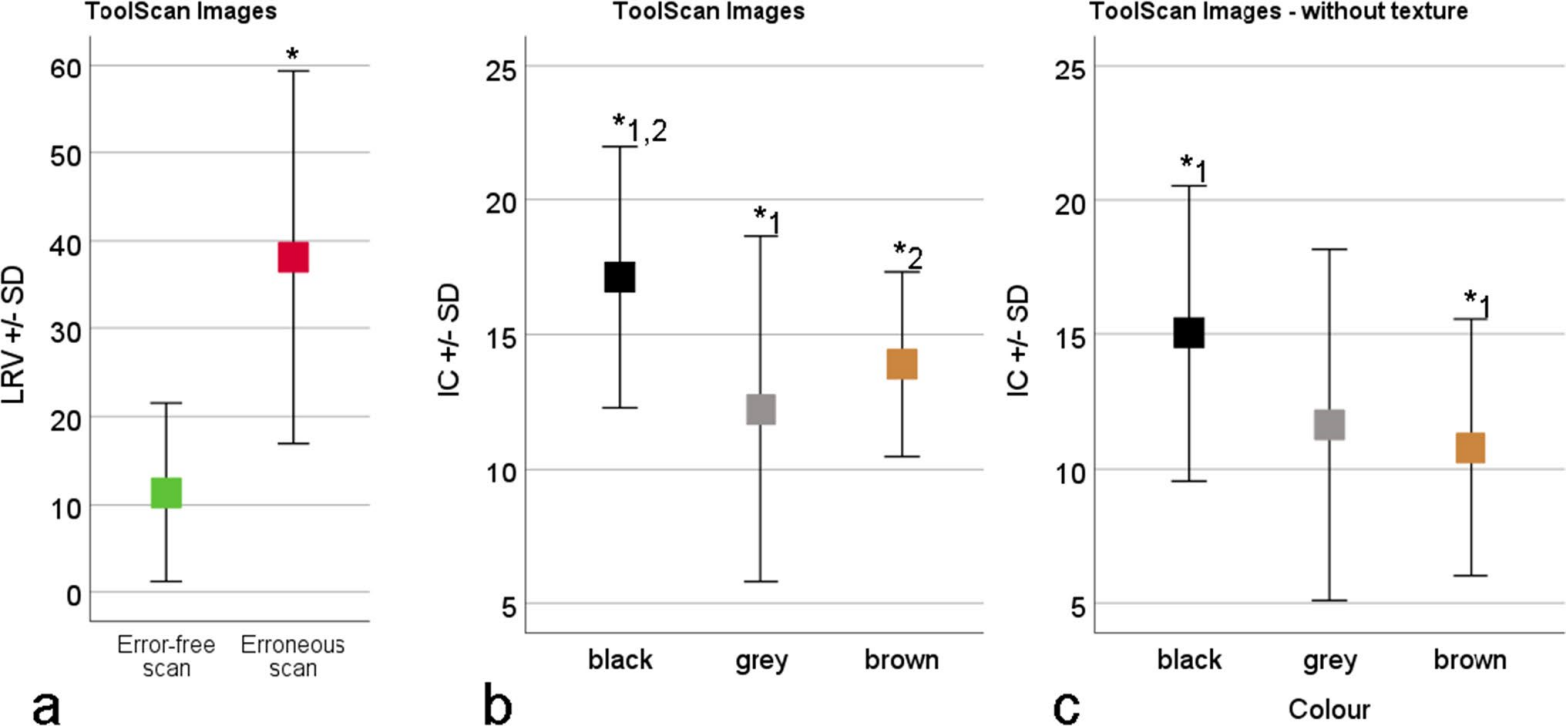
Results of the image contrast (IC) calculation for the ToolScan 3D scanning device. (**a**) The effect of the light reflectance value (LRV) to the scan quality. The casting materials leading to erroneous scans show significantly* higher LRV values. (**b**) The effect of the colour on the IC for the images with texture. Black materials show significantly* higher IC than grey or brown. (**c**) The effect of the colour on the IC for the images without texture. Black materials show significantly* higher IC than brown

For the ToolScan an effect of the LRV on the possibility for scanning the material could be detected (Fig. 12a). Furthermore, no correlation of the IC with the LRV for either the images acquired with or without texture (*P* > 0.05) could be found. When analyzing the images with texture, we detected an effect of the colour (Fig. 12b). The IC value of the black materials was significantly higher than the value of the grey (*P* = 0.04) and brown (*P* = 0.047) materials. For the images without texture, the black samples showed significantly higher values (*P* = 0.04) than the brown samples (Fig. 12c). No other effects could be detected on the ToolScan results.

### Quality and consistency of cut marks in the test materials

**Fig. 13:**
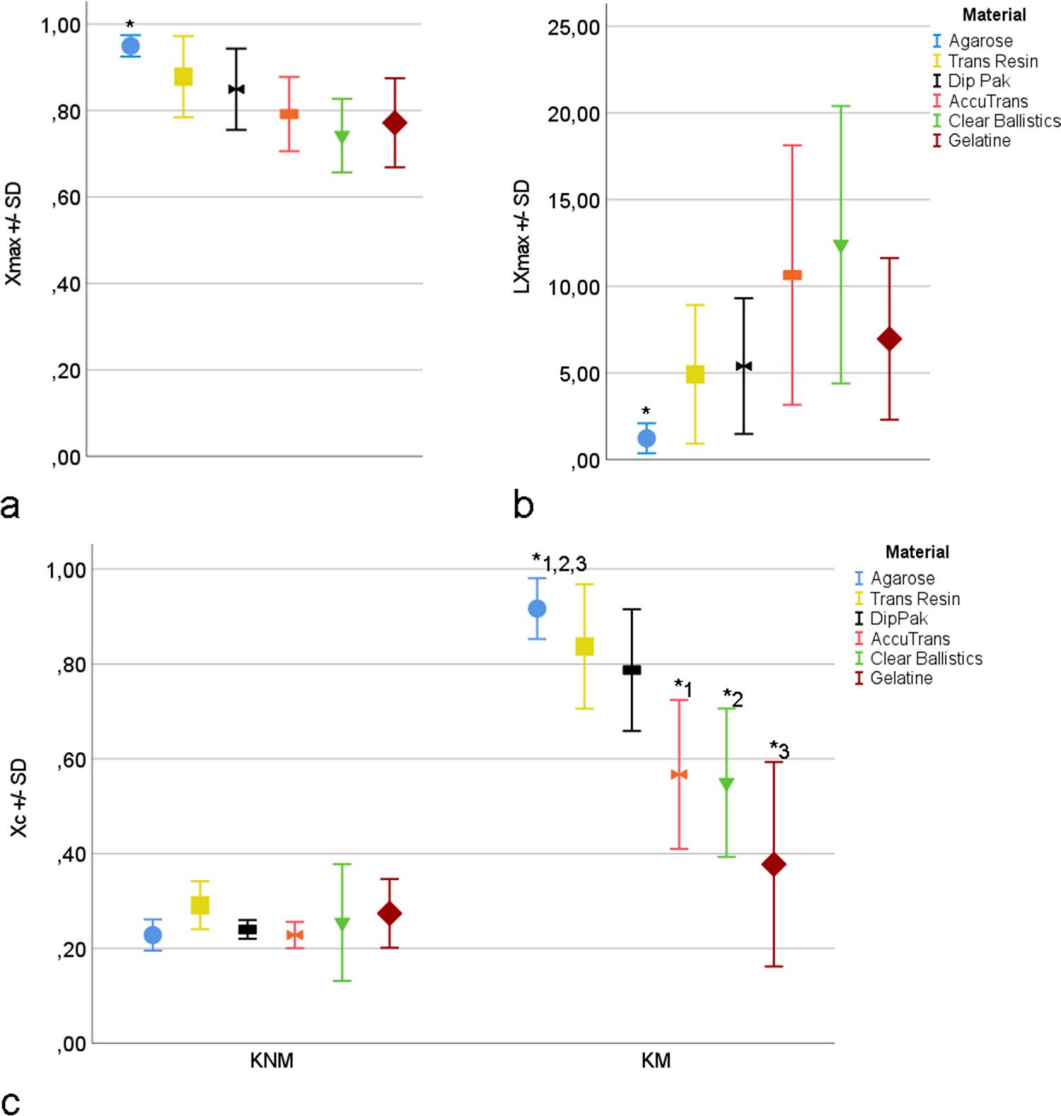
The effect of the test material. Agarose shows significantly* higher values for X_max_ (**a**) and lower values for L_Xmax_ (**b**). The values of the X_C_ for known matches (KM) of agarose are significantly higher than for AccuTrans®, Clear Ballistics™ and gelatine. For the known non-matches (KNM) the values for X_C_ do not differ significantly

The analysis of the results of the mean cross-correlation coefficient X_max_ showed that the value for agarose of 0.95 + / − 0.025 was significantly higher than that of all other tested materials (Trans Resin: 0.88 + / − 0.10, Dip-Pak®: 0.85 + / − 0.10, AccuTrans®: 0.79 + / − 0.09, Clear Ballistics™: 0.74 + / − 0.09, gelatine: 0.77 + / − 0.10; all *P* = 0.00; Fig. 13a). Furthermore, agarose was found to have the lowest respecting lags L_Xmax_ of 1.23 + / − 0.87, significantly lower than the values of all other materials (Trans Resin: 4.92 + / − 4.00, Dip-Pak®: 5.4 + / − 3.9, AccuTrans®: 10.65 + / − 7.49, Clear Ballistics™: 12.40 + / − 8.00, gelatine: 6.97 + / − 4.67; all *P* = 0.00; Fig. 13b).

The analysis of the cross-correlation coefficients X_C_ of the signatures of the KM revealed the highest value for agarose (Fig. 13c) with 0.92 + / − 0.06, significantly higher than the results for AccuTrans® (0.57 + / − 0.16, *P* = 0.00), Clear Ballistics™ (0.55 + / − 0.16, *P* = 0.00), and gelatine (0.38 + / − 0.22, *P* = 0.00). Compared to Trans Resin (0.84 + / − 0.13) and Dip-Pak® (0.79 + / − 0.13), no effect could be detected (*P* > 0.05).

In the analysis of the cross-correlation coefficients X_C_ of the KNM of all materials (AccuTrans®: 0.23 + / − 0.03, agarose: 0.23 + / − 0.03, Clear Ballistics™: 0.25 + / − 0.12, Dip-Pak®: 0.24 + / − 0.02, gelatine: 0.27 + / − 0.07, Trans Resin: 0.29 + / − 0.05), no significant differences were found.

The comparison of the cross-correlation coefficients X_C_ for the KM with the KNM of all materials revealed significant differences for AccuTrans®, agarose, Clear Ballistics™, Dip-Pak®, and Trans Resin (all *P* = 0.00). For gelatine no significant difference could be detected (*P* = 0.32).

### Elastic properties of the test materials

The indentation test revealed Young’s Moduli E_i_ for gelatine: 0.12 + / − 0.04 MPa, Clear Ballistics™: 0.15 + / − 0.01 MPa, AccuTrans®: 1.23 + / − 0.11 MPa, agarose: 1.39 + / − 0.12 MPa, Dip-Pak®: 2.69 + / − 0.05 MPa, and Trans Resin: 4.01 + / − 0.20 MPa.

## Discussion

The examination of tool marks on human tissues and, in particular on bone and cartilage, is gaining increasing importance in the field of tool marks. However, only limited research has been carried out to date on basics of the methodology such as sample preparation, casting, and test mark materials. Our intention with this work was to obtain valuable information that can positively influence the effectiveness of these examinations and the quality of the results.

We conducted cleaning experiments on 75 porcine cartilage samples contaminated with porcine blood and fat. In further studies, comparable experiments should also be performed on human tissue. In our experiments, the cleaning method *Triple Casting* yielded significantly higher scores than the other methods (all *P* < 0.05) and seems, therefore, recommendable. Furthermore, casting is the most uncomplicated of the tested methods, and in the context of an examination, taking casts is a necessity anyway which does not require much additional effort. In this study, we used AccuTrans® AB brown, an addition-reaction silicone elastomer for the cleaning experiments. Since a large number of casting materials with differing properties (such as viscosity or curing time) exist, comparable experiments should be carried out in further studies with different casting materials. It should also be explored, whether the crosslinking mechanism of the casting materials (by addition or condensation) has an influence on the applicability to cartilage tissue, which was not part of this work.

With the aim of increasing the quality of the casts of marks in costal cartilage, the emergence of the grain-like dots was investigated. Furthermore, we conducted experiments aiming to avoid the occurrence of dots. The experiments on 36 cartilage samples with formaldehyde, paraformaldehyde and glutaraldehyde have shown that fixing the tissue did not reduce the dots. Further studies are needed to explore whether it is possible to avoid the dots by using other fixatives or methods. Due to the limited amount of body donors, porcine cartilage was used for the cleaning and dots experiments. Further research is needed to determine whether these results can also be applied to human cartilage tissue.

The results of the comparative study of the diameters and distribution of dots and chondrocytes strengthen the assumption that the dots are casts of the lacunae, the vacancies in which the chondrocytes are located in the extracellular matrix of the cartilage tissue. The occurrence of the dots, some isolated and some in columns, resembles the distribution pattern of chondrocytes in human rib cartilage, which occur scattered and in isogenic groups. The way in which the casting material penetrates into the lacunae was not investigated in this study. It appears possible that the chondrocytes are displaced by the heavier casting material. It is also a possibility that the chondrocytes were already destroyed when the tissue was cut, leaving the lacunae empty. Because of a limited number of body donors, measurements of dots were only performed on 3 female samples and measurements of chondrocytes were performed only on 4 female samples. Despite this limited sample size, we believe that the results support the hypothesis that the dots are casts of the lacunae. No other structure with similar properties (size, geometry and distribution) exists in the cartilage tissue. Nevertheless, further studies are necessary to confirm this assumption.

Another basis of high-quality casts is the selection of the appropriate material. For this purpose, we have investigated the image contrast IC of 31 casting materials under the light microscope and the tool marks scanner, as it is a crucial factor. Striated marks are usually examined under oblique light. The contrast then results from the brightness values of the striations illuminated with different intensities and those lying in the shadows.

Under the light microscope, all blue and green samples were excluded as no usable image with a well-defined striation pattern could be seen. It seems likely that a low opacity of these materials is the reason for this effect. This was not assessed in this work and should be investigated in further studies. It was also found that black materials did achieve the lowest IC results under the light microscope. We have determined the contrast from the standard deviation of the brightness values of the pixels per image. The reflective intensity I_R_ of a surface illuminated with the intensity I_I_ is calculated according to Eq. (4) [47] in which λ is the angle of incidence of the light rays and Τ represents the degree of reflection. The wider the difference between I_R_ of the brightest and darkest pixels, the higher the contrast values that can be achieved. For dark materials, this difference is smaller by comparison, since even with maximum illumination (angle of incidence 90°), the maximum achievable brightness value is lower than for brighter materials with minimum illumination (angle of incidence 0°) I_R_ = 0 for all materials. However, the results of this study show higher results for brown materials than for the brighter white materials. This is likely due to various factors. A lower opacity of the brighter materials could lead to striations being translucent. The diffuse reflection of the illuminated striations could also have an influence. Both effects lead to a brightening of the striations lying in the shadow, thus reducing the contrast. The possibility of diffraction effects at the edges of the striations could also have a negative influence.

4 $${I}_{R}={I}_{I}*\left|sin(\lambda )\right|*\tau$$

On the ToolScan, 16 of the 31 samples were excluded. An analysis of the LRV showed that the included samples have a significantly lower LRV (*P* = 0.00) than the excluded samples. The scan data of the ToolScan is generated from laser scanning and the image data created from a multitude of images illuminated by ring light. Obviously, materials with comparatively high LRV, like white and light grey, are more likely to lead to incorrect measurements than darker shades. At which point in the scanning process the false measurements occur was not considered. The measurement results suggest that the use of very dark or black impression materials is generally preferable for ToolScan. Light-coloured casting materials have shown to be less suitable.

In our study, we did not address the applicability of the casting materials to cartilage tissue. This issue should be considered in a future work.

A tool marks examination can only be successful if a suitable test material has been selected. For stab and cut marks, it is important that the material has the ability to reproduce even very fine grooves. In this work, we compared 6 elastic materials regarding their quality. For this purpose, cut marks were created and cast and their surface was scanned. The scan data was divided into individual signals and these were aligned with each other by cross-correlation. The cross-correlation coefficients X_max_ calculated in this process and the associated lags L_Xmax_ can be regarded as a measure of the quality of the material.

The analysis of the mean lags L_Xmax_ calculated when aligning the signals of individual marks showed that the marks generated in agarose had the lowest value, i.e. the least shifting was required when aligning the signals. In addition, the individual signals showed the highest cross-correlation coefficients X_max_ when aligned with each other and, thus, the highest degree of matching compared to all other materials tested in this study. Both results suggest that cut marks in agarose have the highest consistency compared to all other materials tested, which is an important factor for a test material.

Another important demand on the material is that it must be possible to reproduce marks in the test material. In addition, similarities and differences must be clearly recognizable. To verify these attributes, we generated known match (KM) and known non-match (KNM) cut marks and also cross-correlated them. Our results show that of all the tested materials, marks in agarose achieved the highest cross-correlation coefficients for KM and, together with AccuTrans®, the lowest coefficients for KNM. Our results of the cross-correlation coefficients of agarose for KM (0.92 + / − 0.06) and KNM (0.23 + / − 0.03) are comparable to the studies on the striated marks of screwdrivers of Baiker et al. [36, 37] who calculated slightly higher coefficients for KM of 0.97 + / − 0.01 and similar coefficients for KNM of 0.22 + / − 0.13 for screw driver marks. For all materials except gelatine, the mean values for KM and KNM were significantly distinguishable. However, compared to all other materials, the distance between these two values was the highest for agarose, allowing the best possible discrimination between KM and KNM.

During the production of the test cuts, it was, furthermore, noticed that agarose can be cut with only little force. Agarose belongs to the hydrogels and we assume that the water contained in the material lubricates the knife blade, thus reducing friction and the force required. This effect also reduces the risk of accidents during sample preparation, especially compared to the firmer materials such as Trans Resin and Dip-Pak®. In addition, the reduced friction is likely to prevent chatter marks and friction-induced abrasion on the cut marks, thus, being a reason for the high quality of the marks in agarose. All results combined allow us to conclude that, of the materials tested here, agarose is the most promising test material for stab and cut marks in cartilage. Moreover, the material is relatively inexpensive and non-hazardous. The production of agarose sheets is also uncomplicated, and it is available in different shapes and thicknesses.

**Fig. 14:**
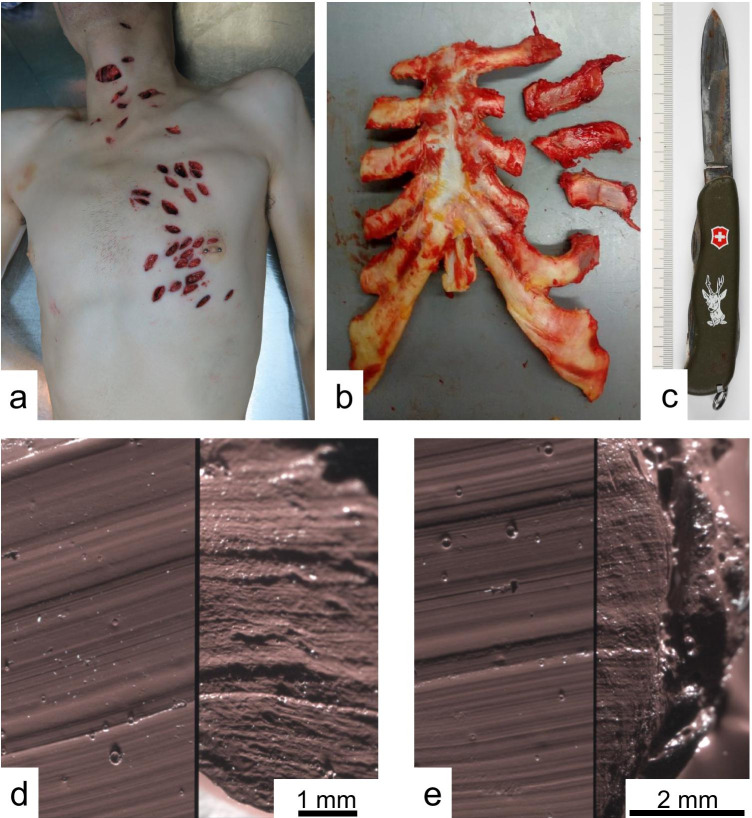
Case work of a homicide. (**a**) The 27-year-old male victim died of multiple stab wounds to his body; additional injuries included blunt trauma to the head and back. (**b**) Three of the stab wounds to the chest severed the left third, fourth, and fifth ribs in the costal cartilage and left evaluable tool marks. (**c**) The suspected murder weapon was a Swiss Army knife. (**d**) The microscopic comparison of the stab mark in the third rib (left side of the image) and (**e**) the fifth rib (left side of the image) with the test mark of the knife made in agarose (each right half of the images) revealed for both marks a sufficient number of matching striations leading to the identification of the knife as the murder weapon

The results of the indentation tests reveal Trans Resin to achieve the highest Young’s Modulus of 4.01 + / − 0.20 MPa. In a previous work, we performed indentation tests on human costal cartilage samples and calculated a Young’s Modulus of 14.55 + / − 6.59 MPa [40] which is close to four times higher. Although these values differ so considerably, we successfully used agarose sheets in a casework of stab marks in human rib cartilage and have been able to identify the instrument of crime through test marks produced in the material (Fig. 14). This leads to the conclusion that the Young’s Modulus, by itself, is not a sufficient parameter for defining the elastic properties of a suitable test material for rib cartilage. We hypothesize that the quotient of Young’s Modulus and tensile strength of test material and rib cartilage must be similar to produce comparable stab or cut marks in both materials. The Young’s Modulus represents the elasticity of the material while the tensile strength defines the stress level at which the material ruptures. Upon contact with a cutting edge, an elastic material initially undergoes elastic strain until the stress on the surface exceeds the tensile strength and the material is cut, which can be viewed as a tearing at the microscopic level. In future studies, this aspect should be investigated further as it may also have implications for other tool marks disciplines such as the examination of knife cuts in tires.

With the results of this work, we provide valuable insights to enhance the examination methods of tool marks in general and on human tissue or costal cartilage in particular.

## Data Availability

Not applicable.

## Footnotes

1 Hans Gross: Born 26 December 1847 in Graz (Austria), died 9 December 1915 in Graz. Judge; jurist, criminologist, founder of the Graz school of criminology. [Source: http://lithes.uni-graz.at/handbuch/gross_hanns.html, as of 01/2021].

2 Richard Kockel: Born 05 January 1865 in Dresden (Germany), died 19 January 1934 in Leipzig. Prof. of Forensic Medicine at the Medical Faculty of the University of Leipzig. [Source: https://research.uni-leipzig.de/catalogus-professorum-lipsiensium/leipzig/Kockel_188/, as of 01/2021].

3 Dip-Pak®. Manufacturer: Fidelity Chemical Products (USA). Product Name: Type I Transparent Coating.

4 Trans Resin bone cutting plates. Distributor: BVDA International BV (NL). Manufacturer: Intercontinental Chemical Products Limited (UK). Product Name: Protecta-Cote.

5 Clear Ballistics®. Manufacturer: Clear Ballistics (USA). Product: 10% ballistic gelatin air rifle block.

6 tween® 20. Manufacturer: Merck KGaA (Germany), EMD Millipore Corporation (USA).

7 AccuTrans® AB brown. Manufacturer: Coltène/Whaledent Inc. (USA).

8 The software application of the ToolScan device can display the scan data as a pure representation of the height information (no texture) or additionally with the brightness information of the camera unit (with texture).

9 Agarose Standard ROTI® Garose for DNA/RNA electrophoresis. Manufacturer: Carl Roth GmbH + Co. KG (Germany).

10 GELITA® BALLISTIC/ORDNANCE GELATINE. Manufacturer: GELITA AG (Germany).
